# Principles of cartilage tissue engineering in TMJ reconstruction

**DOI:** 10.1186/1746-160X-4-3

**Published:** 2008-02-25

**Authors:** Christian Naujoks, Ulrich Meyer, Hans-Peter Wiesmann, Janine Jäsche-Meyer, Ariane Hohoff, Rita Depprich, Jörg Handschel

**Affiliations:** 1 Clinic for Maxillofacial and Plastic Facial Surgery, Westdeutsche Kieferklinik, University of Düsseldorf, Germany; 2 Clinic for Cranio-Maxillofacial Surgery, University of Münster, Germany; 3 Clinic for Orthodontics, University of Münster, Germany

## Abstract

Diseases and defects of the temporomandibular joint (TMJ), compromising the cartilaginous layer of the condyle, impose a significant treatment challenge. Different regeneration approaches, especially surgical interventions at the TMJ's cartilage surface, are established treatment methods in maxillofacial surgery but fail to induce a regeneration *ad integrum*. Cartilage tissue engineering, in contrast, is a newly introduced treatment option in cartilage reconstruction strategies aimed to heal cartilaginous defects. Because cartilage has a limited capacity for intrinsic repair, and even minor lesions or injuries may lead to progressive damage, biological oriented approaches have gained special interest in cartilage therapy. Cell based cartilage regeneration is suggested to improve cartilage repair or reconstruction therapies. Autologous cell implantation, for example, is the first step as a clinically used cell based regeneration option. More advanced or complex therapeutical options (extracorporeal cartilage engineering, genetic engineering, both under evaluation in pre-clinical investigations) have not reached the level of clinical trials but may be approached in the near future. In order to understand cartilage tissue engineering as a new treatment option, an overview of the biological, engineering, and clinical challenges as well as the inherent constraints of the different treatment modalities are given in this paper.

## Introduction

Skeletal defects in the adults craniofacial skeleton compromises mainly bony structures, whereas chondral or osteochondral defects are less common, but when present are accompanied by a significant morbidity. Articular cartilage tissue is present in the adult patient in the temporomandibular joint (TMJ). Despite this relative minor prevalence of cartilage defects towards bony destructions, defects of the TMJ plays an important clinical role in maxillofacial surgery [1]. The consequences oft TMJ tissue alteration may be pain and functional impairments. Disturbances in the cartilage layer are often associated with severe functional disturbances and a subsequent progression of cartilage degeneration or inflammation. Diseased or lost TMJ structures are most common as sequelae of trauma, degeneration, infection, or autoimmune disease. The treatment of TMJ defects is complex and based mainly on the underlying cause of defect generation [2]. Indications for a surgical management can be devided in relative and absolute indications. Due to the multitude of pathogenic disturbances and based on the extent of TMJ structure involvement attempts to heal TMJ lesions span the whole range between symptomatic measures and extensive surgical interventions. Absolute indications are commonly reserved for more severe alterations of the TMJ disc or the condyle. Whereas interventions at the base of the skull are seldom performed, repair of the disc or the condyle is a matter of special interest in maxillofacial surgery. The spectrum of surgical procedures for the treatment of temporomandibular joint disorders is wide and ranges from simple arthrocentesis and lavage to more complex open joint surgical procedures. The most invasive procedure is the resection and reconstruction of the TMJ. Autologous cartilage-bone grafts, e.g. from the rib, and alloplastic materials like a patient-fitted prostheses can be used for the reconstruction of the joint. The issue on engineering the TMJ disc, reviewed extensively by Allan and Athanasiou [3], is from a structural and biological aspect distinct from those at the cartilage containing condylar head [4].

As articular cartilage has, in contrast to bone, only a limited capacity to regenerate itself, regeneration supporting therapies are of high relevance when this tissue is involved in the destruction process [5]. It is well known that lesions which are confined to the articular cartilage alone have little or no capacity to heal. In general, the patients become symptomatic and a significant progression to osteoarthritis is possible [6]. Those lesions that penetrate the subchondral bone have a limited repair capacity because they have access to the bone marrow space and chondroprogenitor cells. The regeneration and repair of lesions in the condylar head depend therefore on the extent of destruction and, when being severe, impose a significant problem in maxillofacial practice. That is why new therapeutic strategies focus on cartilage tissue engineering strategies to regenerate or reconstruct condylar cartilage [4,7]. As an unimpaired biomechanical function of articular cartilage containing joints is dependant on the anatomical integrity of the joint [8], custom made engineered structures are of importance [9]. As cartilage defects are typically seen in arthrotic or arthritic patients, cartilage engineering may be today of special relevance in these patient groups but may be in future also used to repair more complex cases.

It is important to note that in contrast to maxillofacial surgery, where recently the economically most important skeletal tissue substitute is bone, cartilage plays the most prominent role in orthopaedics [10]. Cartilage engineering therapies were mainly invented and tested in the orthopaedic field but are now introduced in maxillofacial surgery. Based on a multitude of valuable basic scientific, pre-clinical as well as clinical studies, advances have been made in all fields of cartilage tissue engineering. The review is intended to give an updated overview of cartilage tissue engineering. To understand the evolving field of cartilage engineering it is important to give a brief introduction in cartilage histology and cartilage regeneration and to consider the common repair procedures, before the field of cartilage tissue engineering in the narrower sense is discussed in detail.

## Cartilage histology

The three types of cartilage (hyaline cartilage, elastic cartilage, and fibrocartilage) are present in adults. The type of cartilage differs in the various locations of the body (at the articular surface of bones, in the trachea, bronchi, nose, ears, larynx, and in intervertebral disks). The cartilage of the condylar head is fibroelastic [11]. The histology of the condyle mirrors the functional needs of mandibular movement [12]. The cartilage cap of the joint contains cells, fibers, and amorphous ground substance. It is dominated by the acellular elements and is devoid of blood vessels and nerves. Cartilage is occupied by an extensive extracellular matrix that is synthesised by chondrocytes. A chondrocyte always generates from a mesenchymal cell, the prechondrogenic cell or chondrocyte precursor cell, which is – due to lack of specific markers – only defined by the expectation that its daughter cell will be a differentiated chondrocyte (for review see Behonick and Werb [13]). Chondrocyte precursor cells are of general fibroblastic appearance and synthesises – like fibroblasts – type I and III collagen, fibronectin, and noncartilage-type proteoglycans [14]. Stem cells with chondrogenic potential persist throughout adult life and can be induced to differentiate into chondrocytes during fracture callus formation, osteophyte formation, or as ectopic cartilage.

At its free (superficial) surface, which is contacted by synovial fluid, the chondrocytes are flattened and aligned parallel to the surface (for review see Poole et al. [15]). Below the superficial zone is the midzone where cell density is lower. The ultrastructure of the midzone reveals more typical morphologic features of a hyaline cartilage with more rounded cells and an extensive extracellular matrix. Between this midzone and the layer of calcified cartilage is the deep zone. Deep to the articular cartilage, and separated from it, is a layer of calcified cartilage. The calcified cartilage is not very vascular normally, and the remodeling process is therefore not as effective as in vascularised locations. Cell density is lowest in this zone. The chondrocytes in the calcified zone usually express the hypertrophic phenotype, reaching a stage of differentiation that can also be found in fracture repair. The calcified interface provides excellent structural integration with the subchondral bone. Subchondral trabecular bone is underlying the subchondral plate. The structure and appearance of subchondral bone, being critically dependent on the load situation of the TMJ [16], changes its density by remodelling [17]. The extracellular matrix of fibrocartilage is composed of differentially distributed collagen fibrils and non-collagenous proteins that form an extensive network. Many of the molecules play a structural role, whereas others may be involved in regulating cell function. The ground substance of articular cartilage contains also a large variety of noncollagenous proteins and polysaccharides. The molecules vary in their abundance and structure with anatomical site or the person's age. There are no common features of non-collagenous proteins in respect to their distribution, structure and function. Many of the molecules are proteoglycans, bearing glycosaminoglycan chains, whereas others are glycoproteins or even nonglycosylated proteins.

## Cartilage regeneration

Cartilage is a metabolically active tissue that under normal conditions is maintained in a relatively slow state of turnover by a sparse population of chondrocytes distributed throughout the tissue. Despite the activity of these cells, cartilage has a limited capacity for intrinsic repair, and even minor lesions or injuries may lead to progressive damage (and in case of articular cartilage leads to subsequent joint degeneration) [18-20]. Isolated chondral or osteochondral lesions also may be a significant source of pain and loss of function, and will heal spontaneously only under some circumstances. The repair of cartilage is critically dependent on the extent of tissue destruction. Based on the extent of tissue damage, articular defects can be classified into three types:

- mechanical disruption of articular cartilage limited to articular cartilage

- damage to the cells and matrices of articular cartilage and subchondral bone

- mechanical disruption of articular cartilage and bone

Each type of tissue damage initiates a distinct cell driven repair response [21-23]. The ability of chondrocytes to sense changes in matrix composition and synthesise new molecules are the basis for repair processes [24-27]. The two features that are assumed to play main roles in the limited repair response of articular cartilage are the lack of blood supply and a lack of undifferentiated cells that can promote repair. Chondrocytes can repair defects *ad integrum *in circumstances where the loss of matrix proteoglycans does not exceed what the cells can rapidly produce, if the fibrillar collagen meshwork remains intact, and if enough chondrocytes remain capable of responding to the matrix damage.

The repair and remodeling of osteochondral defects differs from the events that follow injuries that cause only cell and matrix injury or disruption of the articular surface limited to articular cartilage [28]. The extent and outcome of the repair and remodeling responses is critically dependant on the desintegration of the subchondral tissue. Defects that extend into subchondral bone cause, in contrast to superficial defects, bleeding into the defect area. Soon after full thickness defects are present, blood escaping from the damaged subchondral bone forms a hematoma that fills the injury site. The final outcome of the repair tissue typically has a composition and structure intermediate between hyaline cartilage and fibrocartilage, imposing an impaired biomechanical competence. The newly formed tissue is in structure and biomechanical competence different to normal articular cartilage [21,22,24,25,29] imposing decreased stiffness and increased permeability. The impact of load on cartilage structure and function is of outermost importance. Physiologic TMJ loading maintains cartilage structure and function. In the context of articular cartilage repair, it is important to recognise that stresses in a cartilage defect or the surrounding tissue may be altered significantly from their normal mechanical environment, and therefore impairs tissue integrity before and after cell/scaffold implantation.

## Surgical repair strategies

In maxillofacial surgery, there are two general goals for cartilage reconstruction. The first is the immediate need for clinical pain relief and restoration of joint function. The second goal is to prevent or at least delay the onset of subsequent joint alterations. From a practical perspective, the current objective of articular cartilage repair is to avoid the development of a deformed joint surface [30]. Besides non-surgical therapies that are based on the administration of drugs (non-steroidal antiphlogistics, steroids) and biologicals (hyaluronan), surgical options play a significant role aimed to gain pain relief, to restore joint functionality and to prevent progression of joint destruction, especially in severely altered joints. In some instances drastic measures like total TMJ replacement by TMJ prosthesis are necessary to achieve clinical success, but such measures impose the problem of long term complications (material failure, scull base perforation) especially when used in younger patients. The use of alloplastic materials is therefore a matter of controversy in maxillofacial surgery [1]. Dimitroulis [2] stresses in his review on TMJ surgery the demands of a close adaptation to natural tissues when a long term success is envisioned. Most of the experimental and clinical attempts that have hence been made to restore articular cartilage structure aim at re-establishment of biomechanically competent tissue of an enduring nature [31]. The surgical measures to improve temporomandibular joint structure and function without the use of biologically active substances can be conceptualised as methods to improve the condition of the joint fluids (lavage), to mechanically remove diseased or necrotic superficial chondral tissue (shaving, debridement, laser abrasion) and to gain access to the subchondral bone (abrasion chondroplasty, pridie drilling, microfracture techniques and spongialisation). The underlying reason for lavage or debridement is the removal of inflamed or diseased tissue, whereas the method to gain access to subchondral bone is aimed at initiating a spontaneous healing response. Arthroscopic lavage and debridement are often used to alleviate joint pain. Lavage is mainly performed by arthroscopy. Various other methods like free [32] or vascularised tissue transfer [33] are clinically used, but some of these approaches impose unexpected clinical outcomes [34]. In contrast to the orthopeadic field, where an ankylosis of a joint may be the ultimate treatment ratio for complicated cases, iatrogenic ankylosis seems not to be indicated for the TMJ in any clinical situation.

## Cellular repair strategies

The use of cells or cell-containing devices, considered to be tissue engineering strategies, can be performed by different measures [35-37]. Tissue engineering techniques have seen rapid advances and refinements during the last years. Whereas these techniques have been elaborated mainly by orthopaedics, their principle application refers also to the maxillofacial field. Transplants from either autologous or allogenic origin can be harvested in the form of perichondrial or periosteal tissue and as a bulk osteochondral part. Perichondrial or periosteal autotransplantation as a single procedure has been exploited in a variety of protocols elaborated for the treatment of articular cartilage defects. Other tissue engineering concepts such as autologous chondrocyte transplantation (ACT) delivers chondrogenic precursor cells to the defect site. The basic biological principle behind the use of these cell based techniques is the fact that perichondrial and periosteal tissue as well as isolated cell suspensions (ACT) contains cells that possess a life-long chondrogenic potential. A pool of precursor or adult-type stem cells is assumed to be present in these tissues that render self-renewable capacity and are able to induce tissue healing. Implantation of explanted bulk chondral or osteochondral tissue (mosaicplasty), routinely used in orthopaedic joint and bone surgery but seldom applied in the TMJ region [4], is aimed to repair mid-size chondral or osteochondral defects. Experimental studies revealed that graft material persisted for a short time, however, long-term effects are not extensively evaluated. It was demonstrated by retrospective studies that clinical outcomes were acceptable in sense of improved joint functionality and pain relief. Despite the short-term clinical success, the use of non expanded autografts possess a number of disadvantages. The donor site may experience severe morbidity since the explantation site will loose as much chondral or osteochondral tissue as the diseased implantation site will get. Transplantation of extended cartilage containing specimens (iliac crest, digits) [33] are seldom performed in TMJ surgery due to the significant functional impairment in the harvesting region.

Articular chondrocytes are responsible for the unique features of articular cartilage; hence, it seemed rational to use committed chondrocytes to repair a cartilaginous defect. As cells were demonstrated to impose the ability to be expanded in culture the re-transplantation of *ex-vivo *multiplicated cells (autologous chondrocyte transplantation (ACT)) seemed to be a promising treatment strategy. Over the last decade autologous chondrocyte transplantation has gained much scientific and commercial interests. ACT and its several modifications are the most widespread applications of cartilage tissue engineering. In the clinical use of *in vitro *expanded autologous chondrocytes for cartilage repair the aim seemed to be to have an adequate number of expanded cells to implant and an overlying membrane to avoid cell and matrix loss. Brittberg etal. [38] successfully reported in 1994 on autologous chondrocyte implantation using a monolayer culture system to treat cartilage defects. In this procedure, harvested autologous chondrocytes, expanded in a monolayer culture system were transplanted to an osteochondral lesion which was covered by a periosteal flap. The rationale behind this approach was the finding that chondrocytes can, after harvesting, be isolated by enzymatic digestion and expanded in culture 20 to 50 times the initial number of cells [39]. It is known that cells, cultured in monolayers with serum supplementation in the culture media, commence to dedifferentiate. The dedifferentiated chondrocytes share features of primitive mesenchymal cells and on implantation at high density the *in-vitro *expanded primitive immature chondrocytes imitate prechondrogeneic cell condensation and cartilage formation [40,41]. This findings and the initial report by Brittberg had a high impact on cartilage surgery and was regarded as a breakthrough for cell-based cartilage repair strategies. The United States Food and Drug Administration approved in 1997 the cell technology that uses the patient's own chondrocytes to repair cartilage injuries in the knee [42]. This was the first type of cell technology that was regulated by industry for use in expanding autologous cells for human transplantation. In the U.S.A. and Europe, cell processing in a monolayer culture is now been carried out on a commercial basis. The use of autologous chondrocytes was primarily performed in traumatically damaged knee joints [43]. Based on the sum of the experience gained in orthopaedics, preclinical and clinical studies tended to expand the indications to joints others than the knee. To date ACT is clinically used to treat also non-traumatic cartilage defects (arthrosis, arthritis defects), and to repair complex tissue defects (osteochondral defects) by a combination of bone and cartilage products. As a consequence, ACT is now under investigation as a clinical treatment modality also in TMJ surgery.

Whereas ACT is now routinely done some issues must be stressed. In contrast to the clinical outcome rates, limited information is present on the histogenesis of the cell-driven human repair tissue. Biopsy specimens from grafted areas in individuals obtained after autologous chondrocyte transplantation (in the orthopaedic field) indicated that the ACT procedure helps to build up a tissue with hyaline and fibrocartilage-like features [44,45]. Transarthroscopic biopsy specimens obtained from grafted areas demonstrated in general a heterogeneity throughout the repair tissue. Although beneficial short- or middle-term clinical results were reported on a clinical basis [45,46], the ACT procedure has potential disadvantages, such as the risk of leakage of transplanted chondrocytes from the cartilage defects and an uneven distribution of chondrocytes in the transplanted site [47]. Additionally, ACT transplantation is not able to regenerated larger defects. These limitations explain to some extent the finding of a heterogenous tissue formation in the defect site. To overcome these limitations, further developments focus therefore on the *ex-vivo *growth of a three dimensional cartilage-like tissue, which integrates intimately in the defect site after being implanted. Other possible sources of cells for tissue engineering include beneath autologous cells allogenic and xenogenic cells. Each category can be subdivided according to whether the cells are in a more or less differentiated stage. Various mature cell lines as well as multipotential so-called mesenchymal progenitors have been successfully established [48] in bone tissue engineering approches. Moreover, there are some reports using totipotent embryonic stem cells for tissue engineering of bone [49,50]. Another group of cells, which is a special focus of scientific and clinical studies today, is believed to contain multipotential stem cells which are often called "mesenchymal stem cells (MSCs)" [51,52] or "adult stem cells" [53]. Whereas the situation of determined cells is well known to researchers and clinicians in TMJ reconstruction, not only the origin, but also the destiny and clinical usefulness of MSCs in TMJ surgery has not been entirely resolved to date.

## In-vitro engineering strategies

In order to prevent the loss of chondrocytes after cell implantation (in the case of ACT) and to increase the size of a cellular device, extracorporal tissue engineering techniques were considered an alternative pathway [7]. Extracorporal cartilage engineering requires not only living chondrocytes, but additionally the interaction of two other components: extracellular scaffolds and in some instances growth factors. For engineering cartilage tissue *in-vitro *cultured cartilage cells are cultured as described for the ACT procedure in monolayer to increase the cell number. Later on they are grown on two-dimensional or three dimensional bioactive degradable biomaterials that provide the physical and chemical basis to guide their differentiation and three dimensional assembly [54]. In bioreactors outside the body the cellular device is ideally matured to a cartilage-like tissue. New approaches in extracorporal tissue engineering strategies are aimed to improve chondrocyte cell lines and to fabricate scaffold-free three-dimensional micro-tissue constructs. Whether the cell containing device contains an artificial scaffold or not [4], the construct has to be implanted in the defect site to promote cartilage healing. An appropriate method to gain this scaffold-free three-dimensional micro-tissue might be the micromass technology. Cells are dissociated and the dispersed cells are then reaggregated into cellular spheres. The micromass technology relies to a great extend on the presence of proteinacious extracellular matrix. The extracellular matrix may exert direct and indirect influences on cells and consequently modulate their behaviour. In contrast to conventional monolayer cell cultures, the three-dimensional spheres exert higher proliferation rates and their differentiation more closely resembles that seen in situ [55].

Most chondrocyte transplantation studies have, to date, predominantly focussed on the use of an unselected source of chondrocytes [38]. In the ongoing search to improve chondrocyte cell lines, the use of specific chondrocyte populations are now being considered to investigate whether an improved cartilaginous structure would be generated *in-vivo *and *in-vitro *by these specifically selected populations of determined chondrocytes [56]. As distinct phenotypic and functional properties of chondrocytes across the zones of articular cartilage are present, it seemed reasonable to search for the best source of chondrocyte subpopulations [57]. It was reported in this respect that a combination of mid and deep zone chondrocytes seems to be more suitable for the *ex-vivo *generation of a hyaline-like cartilage tissue. Dowthwaite *et al*. [58], have recently reported on an isolation technique for chondrocytes that reside in the superficial zone of immature bovine articular cartilage. These cells, characterised as determined chondrogenic cells, were shown to allow appositional growth of the articular cartilage from the articular surface [59]. Therefore, when chondrocytes are aimed to generate a cartilage-like structure *ex-vivo*, it seems to be reasonable not to gain full thickness cartilage implants but to use subpopulations of chondrocytes. Separation of cartilage zones after the explantation and before cultivation with a selective subpopulation may provide a tool to improve tissue engineering strategies using determined cells. Phenotypic plasticity was tested by a series of *in-ovo *injections where colony-derived populations of these chondroprogenitors were engrafted into a variety of connective tissue lineages thus confirming that this population of cells have properties akin to those of a progenitor cell. The high colony forming ability and the capacity to successfully expand these progenitor populations *in-vitro *[59] may further aid our knowledge of cartilage development and growth and may provide novel solutions in *ex-vivo *cartilage tissue engineering strategies.

Many attempts have been successfully undertaken to refine procedures for the propagation and differentiation of cells by the use of bioreactors [60] or by the use of precursor cells. The use of stem cells offers new perspectives in cell propagation techniques. At present, adult stem cells are able to differentiate into chondrocyte-like cells which are competent to synthesise a cartilage-like extracellular matrix under *in vitro *conditions. Despite the various advantages of using tissue-derived adult stem cells over other sources of cells, there is some debate as to whether large enough populations of differentiated cells can be grown *in-vitro *rapidly enough when needed clinically. The alternative approach of using embryonic stem cells is advantageous in respect to the nearly unlimited capacity of cell multiplication but the clinical use of embryonic stem cells is restricted through legal and ethical issues. The use of unrestricted somatic stem cells (USSC's) gained through umbilical cord blood seems, from a clinical perspective the most promising stem cell approach to date [61]. These cells can be gained from stem cell banks, individually matched prior transplantation, and transplanted without major medical or legal restrictions. Whereas various problems must be considered as a limitation for the use of stem cells in extracorporal cartilage tissue engineering, the use of USSC's is in the clinical testing phase. Whereas more basic research is necessary to assess the full potential of stem cell therapy to reconstitute chondral defects, such therapies may be one treatment option in the near future. In this respect it is important to note that many basic research and preclinical studies are today directed toward the development of gene therapy protocols employing gene insertion strategies [62].

## Conclusion

Cartilage tissue engineering has seen significant improvements in the basic research field as well as in pre-clinical applications. Whereas a lot of these techniques are routinely used (or at least) have gained entrance in clinical trials in orthopaedic surgery, less acceptance can be found in maxillofacial surgery [63]. This may be based to some extent on the specific requirements in TMJ surgery, but from a biological perspective it can be assumed that it may be approached more often in maxillofacial surgery in the next future.

## References

[B1] Dimitroulist T (2005). The role of surgery in the management of disorders of the Temporomandibular Joint: a critical review of the literature. Part 1. Int J Oral Maxillofac Surg.

[B2] Dimitroulis T (2005). The role of surgery in the management of disorders of the Temporomandibular Joint: a critical review of the literature. Part 2. Int J Oral Maxillofac Surg.

[B3] Allen KD, Athanasiou KA (2006). Tissue Engineering of the TMJ Disk: A Review. Tissue Eng.

[B4] Schek RM, Taboas JM, Hollister SJ, Krebsbach PH (2005). Tissue enginnering osteochondral implants for temporomandibular joint repair. Orthod Craniofac Res.

[B5] Kaufmann MR, Tobias GW (2003). Engineering cartilage growth and development. Clin Plast Surg.

[B6] Lohmander LS (2003). Tissue engineering of cartilage: do we need it, can we do it, is it good and can we prove it?. Novartis Found Symp.

[B7] Almarza AJ, Athanasiou KA (2004). Design charachterisrics for the tissue engineering of cartilaginous tissues. Ann Biomed Eng.

[B8] Stamm T, Hohoff A, Van Meegen A, Meyer U (2004). On the three-dimensional physiological position of the temporomandibular joint. J Orofac Orthop.

[B9] Feinberg SE, Hollister SJ, Halloran JW, Chu TM, Krebsbach PH (2001). Image-based biomimetric approach to reconstruction of the temporomandibular joint. Cells Tissues Organs.

[B10] Meyer U, Wiesmann HP (2006). Bone and cartilage engineering.

[B11] Stratmann U, Schaarschmidt K, Santamaria P (1996). Morphometric investigation of condylar cartilage and disc thickness in the human temporomandibular joint: significance for the definition of ostearthrothic changes. J Oral Pathol Med.

[B12] Luder HU (1997). Frequency and distribution of articular tissue features in adult human mandibular condyles: a semiquantitative light microscopy study. Anat Rec.

[B13] Behonick DJ, Werb Z (2003). A bit of give and take: the relationship between the extracellular matrix and the developing chondrocyte. Mech Dev.

[B14] Von der Mark K, Conrad G (1979). Cartilage cell differentiation: review. Clin Orthop Relat Res.

[B15] Poole AR, Kojima T, Yasuda T, Mwale F, Kobayashi M, Laverty S (2001). Composition and structure of articular cartilage: a template for tissue repair. Clin Orthop Relat Res.

[B16] Kruse-Losler B, Meyer U, Floren C, Joos U (2001). Influence of distraction rates on the temporomandibular joint position and cartilage morphology in a rabbit model of mandibular lengthening. J Oral Maxillofac Surg.

[B17] Hu K, Radhakrishnan P, Patel RV, Mao JJ (2001). Regional structural and viscoelastic properties of fibrocartilage upon nanoindentation of the articular condyle. J Struct Biol.

[B18] Coutts RD, Healey RM, Ostrander R, Sah RL, Goomer R, Amiel D (2001). Matrices for cartilage repair. Clin Orthop Relat Res.

[B19] Roughley PJ (2001). Age-associated changes in cartilage matrix: implications for tissue repair. Clin Orthop Relat Res.

[B20] Burr DB (2004). Anatomy and physiology of the mineralized tissues: role in the pathogenesis of osteoarthrosis. Osteoarthritis Cartilage.

[B21] Buckwalter JA, Mankin HJ (1997). Articular cartilage: I. Tissue design and chondrocyte-matrix interactions. J Bone Joint Surg Am.

[B22] Buckwalter JA, Mankin HJ (1997). Articular cartilage: II. Degeneration and osteoarthrosis, repair, regeneration and transplantation. J Bone Joint Surg Am.

[B23] Buckwalter JA, Brown TD (2004). Joint injury, repair, and remodeling: roles in post-traumatic osteoarthritis. Clin Orthop Relat Res.

[B24] Meyer U, Wiesmann HP, Meyer T, Stratmann U, Szulczewski D, Joos U (2001). Mechanical tension regulates differentiation of chondrocytes to osteoblast-like cells in distraction osteogenesis. Int J Oral and Maxillofac Surg.

[B25] Hollmiund A, Hellsing G (1988). Arthroscopy of the temporomandibular joint. A comparative study of arthroscopic and tomographic findings. Int J Oral Maxillofac Surg.

[B26] Martin JA, Buckwalter JA (2006). The role of chondrocyte-matrix interactions in maintaining and repairing articular cartilage. Biorheology.

[B27] Martin I, Obradovic B, Treppo S, Grodzinsky AJ, Langer R, Freed LE, Vunjak-Nonakovic G (2001). Modulation of the mechanical properties of tissue engineered cartilage. Biorheology.

[B28] Buckwalter JA, Finerman G (1992). Mechanical Injuries of Articular Cartilage. Biology and Biomechanics of the Traumatized Synovial Joint.

[B29] Buckwalter JA, Martin JA, Olmstead M, Athansiou K, Rosenwasser MP, Mow VC (2003). Osteochondral repair of primate knee femoral and patellar articular surfaces: implications for preventing post-traumatic osteoarthritis. Iowa Orthop J.

[B30] Reinholz GG, Lu L, Saris DB, Yaszemski MJ, O'Driscoll SW (2004). Animal models for cartilage reconstruction. Biomaterials.

[B31] Macintosh RB (2000). The use of autogenous tissues for temporomandibular joint reconstuction. J Oral Maxillofac Surg.

[B32] Zhu SS, Hu J, Li N, Zhou HX, Luo E (2006). Autogenous coronoid process as a new donor source for reconstruction of mandibular condyle: an experimental study on goats. Oral Sug Oral Med Oral Pathol Oral Radio Endod.

[B33] Shenaq SM, Klebuc MJ (1994). The iliac crest microsurgical free flap in mandibular reconstruction. Clin Plast Surg.

[B34] Baek RM, Song YT (2006). Overgrowth of a costochondral graft in reconstruction of the temporomandibular joint. Scand J Plast Reconstr Surg Hand Surg.

[B35] Meyer U, Berr K, Wiesmann HP, Kubler N, Handschel J (2006). Cell based bone reconstruction therapies. Principles of clinical approaches. Int J Oral Maxillofac Implants.

[B36] Handschel J, Depprich R, Kubler N, Wiesmann HP, Meyer U (2006). Cell based bone reconstruction therapies. Cell sources. Int J Oral Maxillofac Implants.

[B37] Edwards PC, Mason JM (2006). Gene enhanced tissue engineering for dental hard tissue regeneration. (1) Overview and practical considerations. Head Face Med.

[B38] Brittberg M, Lindahl A, Nilsson A, Ohlsson C, Isaksson O, Peterson L (1994). Treatment of deep cartilage defects in the knee with autologous chondrocyte transplantation. N Engl J Med.

[B39] Wie X, Messnerr K (1999). Maturation-dependent durability of spontaneous cartilage repair in rabbit knee joint. J Biomed Mater Res.

[B40] Anderer A, Libera J (2002). In vitro engineering of human autogenous cartilage. J Bone Miner Res.

[B41] Brittberg M (1999). Autologous chondrocyte transplantation. Clin Orthop Relat Res.

[B42] Hauselmann HJ, Flura T, Marti C, Hauser N, Hedbom E (1998). From chondrocyte culture to joint cartilage replacement. Development of de novo cartilage in vitro. Schweiz Med Wochenschr.

[B43] Vangsness CT, Kurzweil PR, Liebermann JR (2004). Restoring articular cartilage in the knee. Am J Orthop.

[B44] Richardson JB, Caterson B, Evans EH, Ashton BA, Roberts S (1999). Repair of human articular cartilage after implantation of autologous chondrocytes. J Bone Joint Surg Br.

[B45] Peterson LT, Minas T, Brittberg M, Nilsson A, Sjogren-Jansson E, Lindahl A (2000). Two- to 9-year outcome after autologous chondrocyte transplantation of the knee. Clin Orthop Relat Res.

[B46] Minas T (2001). Autologous chondrocyte implantation for focal chondral defects of the knee. Clin Orthop Relat Res.

[B47] Sohn DH, Lottmann LM, Lum LY, Kim SG, Pedowitz RA, Coutts RD, Sah RL (2002). Effect of gravity on localization of chondrocytes implanted in cartilage defects. Clin Orthop Relat Res.

[B48] Yamaguchi M, Hirayama F, Murahashi H (2002). Ex vivo expansion of human UC blood primitive haematopoetic progenitors and transplantable stem cells using human primary BM stromal cells and human AB serum. Cytotherapy.

[B49] Heng BC, Cao T, Stanton LW, Robson P, Olsen B (2004). Strategies for directing the differentiation of stem cells into the osteogenic lineage in vitro. J Bone Miner Res.

[B50] Zur Nieden NI, Kempka G, Rancourt DE, Ahr HJ (2005). Induction of chondro-, osteo-, and adipogenesis in embryonic stem cells by bone morphogenetic protein-2: effect of cofactors on differentiating lineages. BMC Dev Biol.

[B51] Halleux C, Sottile V, Gasser JA, Seuwen K (2001). Multi-lineage potential of human mesenchymal stem cells following clonal expansion. J Musculoskelet Neuronal Interact.

[B52] Pittenger MF, Mackay AM, Beck SC (1999). Multilineage potential of adult human mesenchymal stem cells. Science.

[B53] Moosmann S, Hutter J, Moser C, Krombach F, Huss R (2005). Milieu-adopted in vitro and in vivo differentiation of mesenchymal tissues derived from different adult human CD34-negative progenitor cell clones. Cells Tissues Organs.

[B54] Handschel J, Depprich RA, Kubler NR, Wiesmann HP, Ommerborn M, Meyer U (2007). Prospects of micromass culture technology in tissue engineering. Head & Face Medicine.

[B55] Springer IN, Fleiner B, Jepsen S, Acil Y (2001). Culture of cells gained from temporomandibular joint cartilage of non-absorbable scaffolds. Biomaterials.

[B56] Redman SN, Oldfield SF, Archer CW (2005). Current strategies for articular cartilage repair. Eur Cell Mater.

[B57] Waldman SD, Grynpas MD, Pilliar RM, Kandel RA (2003). The use of specific chondrocyte populations to modulate the properties of tissue-engineered cartilage. J Orthop Res.

[B58] Dowthwaite GP, Bishop JC, Redman SN, Khan IM, Rooney P, Evans DJ, Haugthon L, Bayram Z, Boyer S, Thomson B, Wolf MS, Archer CW (2004). The surface of articular cartilage contains a progenitor cell population. J Cell Sci.

[B59] Hayes DW, Averett RK (2001). Articular cartilage transplantation. Current and future limitations and solutions. Clin Podiatr Med Surg.

[B60] Meyer U, Buchter A, Nazer N, Wiesmann HP (2005). Design and performance of a bioreactor to mechanically promote bone and cartilage tissue formation. Br J Oral Maxillofac Surg.

[B61] Kogler G, Sensken S, Airey JA, Trapp T, Mueschen M, Fedhahn N, Liedtke S, Sorg RV, Fischer J, Rosenbaum C, Greschat S, Knipper A, Bender J, Degistrici O, Gao J, Caplan AI, Coletti EJ, Almeida-Porada G, Muller HW, Zanjani E, Wernet P (2004). A new human somatic stem cell from placental cord blood with intrinsic pluripotent differentiation potential. J Exp Med.

[B62] Evans CH, Robbins PD (1995). Possible orthopaedic applications of gene therapy. J Bone Joint Surg Am.

[B63] Yang C, Wang XD, Qui WL, Cai XY, Ha Q (2001). A experimental study on arthroscopic auricular cartilage transplantation for repair of osteochondral defect of temporomandibular joint. Shanghai Kou Quiang Yi Xue.

